# Sporting Mind: The Interplay of Physical Activity and Psychological Health

**DOI:** 10.3390/sports12010037

**Published:** 2024-01-22

**Authors:** Alexandra Martín-Rodríguez, Laura Augusta Gostian-Ropotin, Ana Isabel Beltrán-Velasco, Noelia Belando-Pedreño, Juan Antonio Simón, Clara López-Mora, Eduardo Navarro-Jiménez, José Francisco Tornero-Aguilera, Vicente Javier Clemente-Suárez

**Affiliations:** 1 Faculty of Sports Sciences, Universidad Europea de Madrid, Tajo Street, s/n, 28670 Madrid, Spain; sandra.martin.rodriguez8@gmail.com (A.M.-R.); lauraaugusta.gostian@universidadeuropea.es (L.A.G.-R.); noelia.belando@universidadeuropea.es (N.B.-P.); josefranciso.tornero@universidadeuropea.es (J.F.T.-A.); 2 Psychology Department, Faculty of Life and Natural Sciences, Nebrija University, 28240 Madrid, Spain; 3 Department Ciencias Sociales Act Fis Deporte & Ocio, Universidad Politécnica de Madrid, 28040 Madrid, Spain; juanantonio.simon@upm.es; 4 Facultad de Ciencias Biomédicas y de la Salud, Universidad Europea de Valencia, Pg. de l’Albereda, 7, 46010 València, Spain; clara.lopez@universidadeuropea.es; 5 Facultad de Ciencias de la Salud, Universidad Simón Bolívar, Barranquilla 080002, Colombia; eduardo.navarro@unisimon.edu.co; 6 Grupo de Investigación en Cultura, Educación y Sociedad, Universidad de la Costa, Barranquilla 080002, Colombia

**Keywords:** sports psychology, neurobiological mechanisms, psychological resilience, therapeutic interventions, mind–body connection, social sports dynamics, exercise addiction, cross-cultural sports perspectives

## Abstract

The symbiotic relationship between sports practice and psychological well-being has, in recent times, surged to the forefront of academic and public attention. The aim of this narrative review is to comprehensively explore the intricate pathways linking physical engagement in sports to its subsequent impacts on mental health and synthesize the multifarious effects of sports on psychological health, offering insights for integrating physical and psychological strategies to enhance well-being. From neurobiological underpinnings to therapeutic applications, this comprehensive manuscript provides an in-depth dive into the multifaceted world of sports and psychology. Highlighting evidence-based interventions, this review aspires to offer actionable insights for practitioners, athletes, and individuals alike, advocating for a holistic approach to mental well-being. This manuscript highlights the profound impact of sports on mental health, emphasizing its role in emotional regulation, resilience, cognitive function, and treating psychological conditions. It details how sports induce neurochemical changes, enhance brain functions like memory and learning, and aid against cognitive decline. This review also notes the benefits of regular exercise in mood improvement, stress management, and social skill enhancement, particularly when combined with mindfulness practices. It underscores the importance of considering cultural and gender perspectives in sports psychology, advocating for an integrated physical–psychological approach to promote overall well-being.

## 1. Introduction

**Figure 1:**
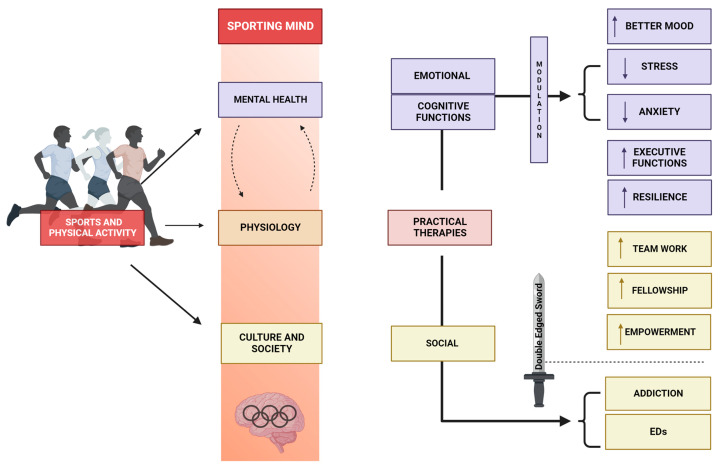
A visual synthesis of the ideas covered and their interconnections in the present review.

Sports possess a distinctive nature as they intricately integrate physical and psychological components, setting them apart as a unique realm of human endeavor. This narrative review aims to comprehensively analyze the intricate mechanisms that link physical engagement in sports to its subsequent impact on mental well-being. Additionally, it seeks to unify the various effects of sports on mental health, offering useful knowledge for incorporating physical and psychological approaches to enhance overall well-being (refer to Figure 1). In this regard, it is a necessity to outline the ancient origins of sports in human culture and their simultaneous growth with psychological understanding [1]. This provides a basis for a more comprehensive examination of the neurobiological pathways, elucidating how physical activity triggers neurochemical interactions that improve and stabilize our mental states [2]. Moreover, the study of emotional regulation and sports examines the therapeutic and harmonizing effects of physical activity [3], while the topic of building resilience focuses on how sports may strengthen the human spirit in the face of life’s challenges [4]. Therapeutic interventions exemplify the clinical integration of sports within the context of healing and rehabilitation.

Regarding mindfulness, any practice may encourage the fusion of a cognitive approach with physical activity [5]. Parallelly, it is needed to explore the relationship between society and sports, which refers to how shared obstacles and successes in sports foster a feeling of camaraderie and interconnection [6]. Additionally, environmental influences investigate the impact of both natural and artificial environments on our psychological interpretation of sports [7]. When discussing the concept of the double-edged sword [8], we acknowledge the adverse effects of sports, such as addiction and fixation. For this last reason, our attention turns to the broader spectrum of human experiences through cultural lenses and gender perspectives. In this context, the interplay between identity and physical exercise reveals diverse impacts on mental well-being. According to this, there are mental strategies to improve performance [9]; thus, we analyze the psychological approaches that improve athletic performance. Then, delving into the practical application of these theories would shed light on new ways of intervening [9]. To summarize, it will synthesize different components into concise and significant insights, highlighting the vital need to integrate physical and psychological approaches to enhance overall well-being in both sports and broader populations.

This work has significant implications for the advancement of sports programs, interventions aimed at mental health, and legislation about public health. A narrative review is necessary due to its interdisciplinary nature, which requires a thorough analytical approach. This approach facilitates a comprehensive examination of the subject matter that goes beyond the constraints of systematic methodologies. It enables meticulous research of the complex interactions among the relevant disciplines.

## 2. Methods

For this review, we conducted a thorough analysis of primary and secondary sources, including scholarly papers, bibliographic indexes, and databases such as PubMed, Scopus, Embase, Science Direct, Sports Discuss, ResearchGate, and the Web of Science. We utilized MeSH-compliant keywords, including Sports Psychology, Neurobiological Mechanisms, Psychological Resilience, Therapeutic Interventions, Mind-Body Connection, Social Sports Dynamics, Exercise Addiction, and Cross-Cultural Sports Perspectives, to search for articles that were published between 2000 and 2023. The subsequent exclusion criteria were employed: (i) works with irrelevant or unrelated issues that are not relevant to the major emphasis of this review; and (ii) doctoral dissertations, conference proceedings, and unpublished works. A team of nine review authors rigorously assessed the titles and abstracts of all gathered publications to determine their appropriateness. Studies that employed obsolete data and addressed irrelevant subjects that did not correspond to the research aims were excluded. The research selection process was conducted by a consistent team of nine review authors, who independently retrieved the relevant data from the chosen publications. This meticulous process ensures the integrity and dependability of the data incorporated in this review. In addition, the review writers engaged in collaborative conversations to integrate the data and deliver a coherent narrative that specifically answers this review’s objectives. The review authors collaborated to combine their experience and views, resulting in a comprehensive and enlightening examination of the literature.

## 3. Historical Overview: Tracing the Evolution of Understanding between Sports and Psychological Health

During the previous Tokyo Olympic Games, which took place in the summer of 2021, gymnast Simone Biles captivated the global public by making a surprise decision to withdraw from five finals. She communicated during a press conference that her primary concern was her mental well-being [10]. In the same year, tennis star Naomi Osaka unexpectedly withdrew from the Roland Garros tournament, citing her struggles with anxiety related to media interactions and the depressive challenges she had been grappling with since her victory in the 2018 US Open [11]. Similar cases have arisen in the experiences of various athletes in recent years, including swimmer Michael Phelps, basketball players Kevin Love and Ricky Rubio, and gymnast Aly Raisman, all of whom serve as a stark reminder of the vital significance of mental health in the lives of elite athletes [12,13]. Simultaneously, numerous studies conducted in recent years have underscored the therapeutic benefits of engaging in physical activity and sports on the mental well-being of children, adolescents, and adults [1,14,15,16].

Currently, there are numerous cases of athletes who are being overstressed and subjected to excessive amounts of ‘unhealthy’ competition, in contrast to many other young adults who are not enjoying the health benefits that participation in physical and sports activities can offer because they are sedentary and not engaged in sports or any form of physical activity. Before delving into the multifaceted analysis of the impact that sports practice has on mental well-being, it is interesting to take a look into the past regarding the evolution of the relationship between sports and psychological health.

The significance of the role that physical activity has played as a part of education and citizen formation can be traced back to the origins of ancient Greece [17]. Philosophers in Greece already advocated the concept of “mens sana in corpore sano”, a sound mind in a sound body, as the symbol of the balance between intellectual development, mental equilibrium, and physical activity, which constitute the core values of the human being [18]. In antiquity, the Greeks were aware of the benefits of physical activity for the comprehensive formation of citizens, as an educational means for both the body and the mind, as well as the psychological equilibrium of the individual [17].

During the Renaissance, there was a resurgence of interest in studies on the connection between physical activity and mental health. In this period, the Italian physician and philosopher Mercuriale (1530–1606) shaped what we could refer to as “medical gymnastics” as a form of preventive medicine, emphasizing the importance of regular exercise. In his book “The Art of Gymnastics Among the Ancients”, published in 1569, Mercuriale laid the foundation for exercise principles based on the works of Greek and Roman authors. This book stressed that all healthy individuals should engage in regular physical exercise, forsake a sedentary lifestyle, and seek, through physical activity, the equilibrium between the body and the mind [18].

The interest in a new approach to education through the significance of physical activity that had its beginnings during the Renaissance reached its culmination in the 18th century during the Enlightenment. Many Enlightenment thinkers during this period devoted their attention to the importance of physical activity and play as tools for improving health and education. However, the foremost among them was Rousseau. In his seminal work “Emile, ou de L’Education”, published in 1762, Rousseau presented the fundamental principles that should underpin the holistic education of children, with a particular emphasis on the importance of physical activity. For Rousseau, physical education becomes a pivotal element in the formation of the youth within a comprehensive pedagogical system that promotes their intellectual development while ensuring their physical and mental well-being [19]. Rousseau’s groundbreaking theories on physical education found widespread acceptance among a multitude of intellectuals, educators, and philosophers during the Enlightenment. Johann Bernhard Basedow attempted to put Rousseau’s educational ideas into practice by establishing the Philantropinum in Dessau, an educational institution where physical education became a fundamental component of youth development. Another example of the impact of Rousseau’s theories during this period can be observed in the work of Pestalozzi, who succeeded in shaping an inclusive and comprehensive educational system that reached the working classes of society and emphasized the importance of physical activity as a key means for both health and personality development [20].

The triumph of bourgeois revolutions and ideological movements such as Romanticism and Nationalism in the 19th century brought about a shift in values and lifestyles for a large segment of the population. During this century, Europe formalized physical education by establishing various national “gymnastic schools” that were closely related to fields such as medicine, pedagogy, and military training. During these years, physical education, along with sports, became a tool that allowed people, especially the youth, to develop their bodies alongside a sound mind, often intertwined with strong nationalist principles [21]. The theories of Guts Muth regarding physical activity and contact with nature for the promotion of health and education served as inspirations for other influential figures. For example, the founder of German gymnastics, Ludwig Jahn, advocated the use of gymnastics as a means to achieve political change through nationalist ideals. In contrast, others like Ling approached gymnastics from a more medical and health-oriented perspective, while figures like Amorós i Ondeano in the French school of gymnastics had a stronger military influence [22].

Simultaneously, in England, Arnold introduced sports activities at Rugby School as a means to instill a set of values and to develop the character and personality of young students. In contrast to those who primarily viewed physical education as a means to enhance physical fitness, a trend emerged during this period that emphasized the importance of physical activity as a tool for facilitating socialization and the education of young individuals. In this line of thought, Wood argued in 1893 that physical education was a crucial means for the holistic development of children, significantly contributing to their emotional and intellectual growth [18].

The contributions of Fechner and Wundt between 1850 and 1860 were instrumental in solidifying psychology as a science. However, the earliest investigations into the relationship between physical activity, sports, and psychology were conducted in both the United States and Europe, leading to a wealth of publications between 1894 and 1900 by authors such as Triplett and Ringelmann [23]. In the early years of the 20th century, scientific research on the connection between physical activity, sports, and mental health expanded significantly, evolving from initial inquiries to institutionalization. The outbreak of the First World War interrupted studies of this nature, but from the 1920s, several significant sports psychology laboratories emerged. These included the one established by Schulten in Berlin, the laboratory created by Coleman Griffith at the University of Illinois, and those developed by Rudik and Puni in the Soviet Union [24,25].

The economic depression of the 1930s and the onset of World War II temporarily pushed research on the influence of physical activity and sports on mental health into the background. However, during these years, some studies did focus on the application of sports and psychology in the military context. These military-oriented investigations have paved the way for the contemporary use of psychology and physical activity in military training [18]. Following the end of the war, research on physical activity and psychological well-being resurfaced and became an area of scientific inquiry and professional practice that continued to evolve throughout the latter half of the 20th century, significantly expanding its scope of knowledge. In 1965, the International Society of Sport Psychology (ISSP) was founded in Rome, and four years later, the European Federation of Sport Psychology (FEPSAC) was established. During this period, a considerable number of specialized journals emerged, with the *International Journal of Sport Psychology* standing out, facilitating the expansion of this field of research.

The connection between physical activity, sports, and psychological well-being has been present since the dawn of humanity. However, this relationship has inevitably been influenced by the political and socioeconomic contexts of each historical period. The establishment of this research area in recent decades, along with the impact these studies have on public opinion, highlights the enduring importance of the numerous connections that exist between sports and psychological health in our modern times.

## 4. Neurobiological Pathways: Deciphering the Brain’s Response to Physical Activity

Currently, it is known that physical activity has modulating effects on cognitive function. Numerous studies show that exercise has the capacity to produce neurochemical and neurophysiological changes in the brain [2,26,27]. It also improves learning processes and cognitive performance, increasing the quality of life and well-being. Regarding neuronal organization as well as cognitive functioning, we now know the positive impact of physical activity on certain brain structures. Systematics reviews such as Heinze et al.’s have repeatedly shown that physical exercise modulates the size and functionality of the hippocampus. Specifically, it was determined that the release of brain-derived neurotrophic factor (BDNF) is facilitated by physical activity [27,28]. BDNF is directly related to the neurogenesis or proliferation of new neurons, protection of neuronal pathways within the hippocampus, and synaptogenesis or facilitation of new connections between neurons [29,30].

Other studies in this line of research showed that people who regularly practice physical exercise have a greater cortical mass [31,32]. A study carried out by Anderson et al. showed that in the early stages of the life cycle, children with sporting habits have a greater volume of grey and white matter. Similarly, these children had a more developed hippocampus and basal ganglia than children with lower levels of activity. In relation to cognitive functioning, children with higher activity levels showed better academic performance and improved memory and executive functions [33]. In relation to learning processes, several studies have shown that physical activity during the school day improves attention, concentration, memory, and thinking processes [34]. All of this has an impact on students’ academic performance [35]. This is possible because physical exercise facilitates the release of neurochemicals such as dopamine, serotonin, and norepinephrine, which are involved in processes such as motivation, mood, attention, perception, memory, and learning [31,36].

Recent research has shown that there is high connectivity between different brain regions in processes as complex as thinking, impulse control, and cognitive flexibility [37]. Structures involved in the control of executive functions associated with the prefrontal cortex are directly linked to regions associated with physical movement [3]. In this sense, it is now known that the cerebellum is not only involved in motor functions but also plays a dynamic role in cognitive function [38]. Specifically, when there is damage to this structure, cognitive processing is affected [39]. Moreover, these positive modulatory effects of physical activity are maintained throughout life [40]. Studies in this line of research showed, through neuroimaging tests, that the hippocampal gyrus and adjacent regions were increased in older adults who practice physical exercise [41,42,43]. This area, which is particularly sensitive to the deterioration associated with chronological age, maintains its plasticity in late adulthood, improving memory and exerting a neuroprotective role [44,45].

In this regard, studies in genetic models of Alzheimer’s disease (AD), such as the THY-Tau22 model, showed that mice who exercised in cages also showed improved performance in activities such as the maze [46,47,48]. This improved performance was consistent with decreased levels of Tau phosphorylation in the hippocampus. In the 3x Tg-AD transgenic model, physical activity facilitated a decrease in oxidative markers as well as in long-term potentiation [49,50]. Subsequent studies in humans showed greater plasticity in the brains of people who engage in frequent physical activity for at least 6 months, increasing connectivity in temporal and frontal areas, which are more vulnerable to cognitive decline associated with aging [51,52]. These results suggest that physical activity is a protective factor against cognitive decline, reintegrating neuronal circuits impaired by aging [53].

At the mitochondrial level, exercise increases the number of muscle mitochondria as well as their content, increasing their volume and enzymatic activity [54]. On the other hand, it also increases the number of copies of mDNA that facilitates a greater number of mitochondria in the muscles, improving their resistance and protecting the tissue during the last stages of the person’s life cycle [55]. It seems to be clear that physical activity has a direct and positive impact on the brain, both structurally and functionally. These results should be considered a therapeutic target in the treatment of various pathologies for which a mainly medical approach is currently maintained. An approach to understanding the neurobiological pathways involved in complex processes such as learning, memory, and executive functions is essential for a multidisciplinary approach to improving cognitive function and preventing cognitive decline.

## 5. Emotional Regulation and Sports: How Physical Activity Modulates Mood and Stress

The integration of regular physical activity and sports into one’s lifestyle is essential for enhancing mental health, as evidenced by numerous studies highlighting their benefits in mood regulation and mental well-being. Mammen and Faulkner’s study underscores this point, suggesting that consistent physical activity can serve as a preventative measure against depression [56]. This beneficial effect is primarily due to the release of endorphins and neurotransmitters like serotonin and dopamine, which are known to improve mood and create feelings of happiness and euphoria [57]. The physiological impact of exercise on mood is both complex and diverse. When individuals engage in physical activity, the body’s endorphin levels rise, leading to what is often termed “feel-good” hormones. These biochemical changes contribute to diminished pain perception and a resultant uplift in mood. Furthermore, serotonin, a neurotransmitter released during exercise, plays a crucial role in regulating mood, appetite, and sleep, with increased levels during and following physical activity leading to a heightened sense of well-being and a decrease in symptoms commonly associated with depression and anxiety [58]. Dopamine, another neurotransmitter released during physical activity, is linked to the brain’s reward and pleasure centers. An increase in dopamine levels can lead to feelings of enjoyment, particularly post-exercise, and contribute to motivation and attention regulation. This is especially beneficial for individuals with mood disorders, where dopamine regulation is often compromised [59].

Furthermore, the social dimension of sports also significantly contributes to improved mood. Participating in team sports or group fitness activities fosters a sense of community and belonging, essential for emotional well-being. The support network provided by these communal activities plays a vital role in reducing feelings of isolation and loneliness, which are often associated with depression and anxiety. It highlights the psychological and social health benefits of participating in sports and physical activity, emphasizing improved social skills, an increased social support, and a stronger sense of community [60]. Additionally, sports and physical activity have been linked to enhanced self-esteem and self-efficacy. Achieving exercise goals, whether related to endurance, strength, or sports skills, contributes to a sense of accomplishment and self-worth, which is particularly important for individuals with a low self-esteem and positively affects their overall mood and quality of life [61].

Hence, the influence of physical activity on mood is profound and multi-dimensional. Through the release of neurotransmitters and endorphins, exercise can directly boost mood and counteract the symptoms of mood disorders. Furthermore, the social and self-esteem aspects of engaging in sports add additional layers of emotional support. These findings emphasize the importance of incorporating regular physical activity as a strategy for maintaining and enhancing mental health. Regarding stress reduction, the role of physical activity is a significant focus in both psychological and physiological research. Sports and other forms of physical activity are known to positively activate the body’s natural stress response. This activation leads to an increased production of norepinephrine, a neurotransmitter key to the brain’s ability to manage stress effectively. Consistent physical activity has been shown to reduce overall tension levels, enhance and stabilize mood, improve sleep quality, and boost self-esteem. A primary mechanism through which exercise contributes to stress reduction is the modulation of the body’s stress hormones. Physical activity decreases cortisol levels, known as the stress hormone, in the bloodstream, alleviating stress and anxiety feelings. Moreover, exercise stimulates endorphin production, brain chemicals that act as natural painkillers and mood elevators. This endorphin increase not only reduces stress but also fosters a sense of well-being [61]. Indeed, exercise provides a diversion from daily stressors. This shift in focus can be a form of mindfulness, allowing individuals to break from the continuous cycle of negative thoughts that often accompany stress and anxiety. The repetitive motions in many exercise forms can be meditative, contributing to mental clarity and calmness [62].

In this line, exercise has an impact on sleep, another critical aspect of its role in stress reduction. Regular physical activity enhances sleep quality, in turn, reducing stress. Exercise increases the time spent in deep sleep, the most physically restorative sleep phase, thus improving overall sleep quality. Better sleep can lower stress levels and improve mental health [63]. To achieve these benefits, Anderson and Shivakumar discovered that as little as five minutes of aerobic exercise can stimulate anti-anxiety effects. This finding is particularly relevant for individuals daunted by long or intense workout sessions, indicating that even short physical activity bouts can contribute to stress reduction, making the idea of exercising more accessible and manageable [64].

Furthermore, regarding emotional regulation and sports, a key aspect is how physical activity cultivates psychological resilience. This resilience, a vital component of mental health, is enhanced through the disciplined, goal-oriented nature of sports. Engaging in sports activities not only provides immediate benefits in mood and stress management but also fortifies individuals against future emotional challenges. By fostering skills such as teamwork, perseverance, and self-efficacy, sports equip individuals with the tools necessary for effectively navigating life’s adversities, thus contributing significantly to their long-term emotional and psychological well-being [64]. This concept is supported by Sarkar and Fletcher’s study, which emphasizes the resilience-building power of sports [65], and the work of Weinberg and Gould, which highlights the role of sports in developing psychological strength and coping strategies [66].

Discipline, a core sports element, educates individuals about dedication, persistence, and disappointment management. These lessons are invaluable for building resilience, as they aid in developing a stronger capacity to handle life’s challenges. The repetitive training nature and commitment to improvement, despite sports setbacks, can translate into a more resilient approach to personal and professional challenges [65]. Teamwork, particularly in team sports, cultivates a sense of community and collective responsibility. Participating in sports teams allows individuals to experience shared success joys and shared failure lessons, enhancing their ability to cope with similar experiences in other life areas. The social support system inherent in team sports acts as a psychological stress buffer, significantly contributing to overall resilience [66]. Goal setting in sports is another resilience-contributing factor. Setting and striving to achieve goals is sports training and competition’s integral part. This process teaches individuals how to set realistic, achievable goals, develop strategies to reach them, and adapt to setbacks. These skills are directly transferable to other life areas, aiding in developing a more resilient mindset in facing life’s challenges [67].

Thus, the multifaceted impact of physical activity and sports on emotional regulation is undeniable. Through the release of neurotransmitters and endorphins, exercise effectively enhances mood and mitigates stress, while fostering psychological resilience and well-being. The synthesis of physiological, psychological, and social benefits demonstrates the essential role of regular physical activity in maintaining and improving mental health. This comprehensive approach not only addresses immediate mood and stress concerns but also equips individuals with enduring tools for emotional resilience, underscoring the profound and lasting significance of incorporating sports and physical activity into daily life.

## 6. Building Resilience: The Role of Sports in Developing Mental Fortitude

Resilience, understood as the ability to functionally and effectively face challenges and difficulties in various contexts [4,68], is a non-innate skill that is built and developed progressively throughout individuals’ life trajectories [69]. Resilience allows individuals to adapt optimally to their environment [70]. “Resilient individuals” are those who, in the presence of stressors, tend to exhibit higher positive coping responses [71,72], lower avoidant responses, and/or anxious responses [73]. It is also associated with higher indicators of overall psychological well-being [74,75] as well as better physical health [76,77,78]. Referring to the psychological resilience theory [79], resilience is the result of individuals’ positive evaluation of stressors, enabling them to generate an emotional and behavioral response in line with their values, goals, and objectives [77]. In this sense, cognitive factors and internal dialogue are particularly important in resilient capacity. Individuals need to perceive stressors as opportunities and challenges to face [80,81]. It is crucial to consider that the positive or negative signs attributed to stressors (and consequently, their subsequent responses) are a complex and cumulative evaluative process influenced by the experiences individuals have been exposed to throughout their life trajectories [82].

In addition to these elements, the literature highlights other variables in understanding resilience that are associated with a greater predisposition to interpret stressors in terms of opportunity. In this regard, “open-mindedness” and its two components (open-mindedness and openness to experience) are personality traits associated with resilient responses [83]. From motivation theories, resilience is also linked to intrinsic motivation [73,84]. Individuals who mobilize their resources motivated by personal satisfaction, enjoyment of the task itself, and intrinsic achievement tend to demonstrate higher positive coping abilities or resilience than those oriented towards external approval, improvement relative to their reference group, etc. [85]. Sports environments, given their nature, are contexts with high stress capacity for individuals, whether in competition or training, pushing both physical and psychological resources to their limits for physio sport improvement [86]. This makes them ideal contexts for training and developing positive and functional coping abilities [86], as well as studying the differential consequences for individuals with different resilient profiles [86]. Hence, it has become an area of interest in studies related to psychology and sports performance in recent decades.

The literature shows that young people who regularly participate in sports activities are significantly more resilient than those who do not participate [87,88]. This positive relationship is maintained when analyzing resilience levels among athletes with different competition levels, with athletes participating in higher-level sports competitions showing greater resilience [89]. This highlights the positive impact of early and continuous exposure to sports challenges on the development of positive coping skills. Regarding factors that enable resilience development in sports contexts, according to the model of Galli and Vealey, resilience is the result of continuous exposure to stressful situations (e.g., training, competitions, handling errors, defeats, seeking technical improvement, etc.) combined with sociocultural influences (coaches, peers, family, regulations, etc.) and personal resources [90]. According to these authors, the “reintegration” process after an adverse event allows for the development of resilient skills. In other words, athletes voluntarily facing self-improvement challenges in a psychosocial context, where “error” is understood as a necessary and inevitable step for improvement, facilitates interpreting stressors in terms of opportunity and challenge. In this sense, continuous exposure to these situations promotes that a positive response becomes a consolidated automatic choice at the cognitive level [86,88].

Alongside the sports practice process, the role models accompanying the athlete (coaches and family) are crucial for the development of this capacity, especially during the early development stages [73,84,90]. Childhood and adolescence are critical periods for learning socially appropriate values [91]. Participation in planned, structured, and repetitive physical activities, carried out according to organized and individualized parameters, such as in grassroots sports, sports schools, sports improvement programs, etc., provides significant benefits for human development in general. Particularly during these stages, young people not only cultivate healthy habits but also influence their internal understanding, social skills, and the formation of their future goals [23]. In the context of participation in physical sports activities, resilience development also occurs through the role played by the teacher/coach as a role model [92]. In grassroots sports structures, the formative work of coaches not only has an impact on sports outcomes but also on the construction of the individual and how they perceive and interpret their internal and external worlds. This was evident in a study conducted by López-Gajardo et al., with young athletes showing a positive relationship between the coach’s transformational leadership and resilience characteristics and a negative association with vulnerability under pressure [93]. In their ability to identify and teach desirable skills and behaviors, the values coaches possess can and should shape, guide, and change the behaviors and values of young athletes [94]. When it comes to adult athletes, Sarkar and Hilton found that coaches continue to have a positive impact on the development of athletes’ resilience when they establish a strong relationship between them, create a challenging yet facilitating environment, and serve as examples of resilient coping in training and competitions [92].

## 7. Therapeutic Interventions: Sports Practices in Clinical Settings

The utilization of sports practices as therapeutic interventions in clinical settings has garnered significant attention in the realm of mental health treatment. This integration of sports-based activities offers a unique and often effective approach to addressing various psychological conditions. The fundamental rationale behind using sports as therapeutic interventions lies in the numerous psychological benefits that physical activity offers. Regular engagement in sports has been associated with reduced symptoms of anxiety and depression, enhanced mood, and improved self-esteem [95]. Furthermore, the structure and social aspects of sports provide additional therapeutic benefits, including improved social skills, an increased sense of community, and better coping strategies for stress and adversity [96].

### 7.1. Sports Therapy in Treating Mental Health Disorders

Sports therapy has emerged as a crucial intervention in the treatment of various mental health disorders. This approach is particularly effective for some of the most prevalent mental health issues today. Below are the five significant mental health disorders where sports therapy has shown considerable efficacy, backed by empirical research. The following mental health disorders represent some of the most common and impactful conditions affecting populations worldwide. Major depressive disorder (MDD) is a leading cause of disability, affecting approximately 264 million people globally, which accounts for nearly 3.4% of the world’s population [97]. Anxiety disorders are also widespread, impacting about 3.8% of the global population, with a higher prevalence among women and younger individuals [98]. Post-traumatic stress disorder (PTSD) affects approximately 3.9% of the world’s population at some point in their lives [99]. Attention deficit hyperactivity disorder (ADHD) is estimated to affect about 2.2% of the global adult population [100]. Finally, bipolar disorder impacts around 1% of the global population, illustrating its significant presence across different regions [101]. These statistics underscore the extensive reach of these mental health issues, highlighting the critical need for effective therapeutic interventions such as sports therapy.

#### 7.1.1. Major Depressive Disorder

MDD stands as a formidable global health challenge, significantly impacting daily functioning and quality of life. The pioneering study by Blumenthal et al. has been instrumental in elucidating the therapeutic benefits of physical activity in managing MDD. Their research compellingly demonstrated that regular physical exercise could effectively mitigate depressive symptoms, comparable in efficacy to antidepressant medications [102]. This finding not only underscores the potential of sports as a non-pharmacological therapeutic option but also highlights the broader benefits of exercise for mental health.

The efficacy of exercise in treating depression extends beyond the immediate alleviation of symptoms. A study by Schuch et al. reinforced this notion, revealing that physical activity can impart long-term mood enhancement and resilience against future depressive episodes. This study suggested that the combination of physical, psychological, and social benefits derived from exercise contributes to its overall effectiveness in treating MDD [103]. Additionally, the role of exercise in modifying brain chemistry provides a biological basis for its therapeutic impact. A study by Mikkelsen et al. demonstrated that exercise could stimulate neuroplastic changes in the brain, enhancing neural growth, connectivity, and overall brain health. This neurobiological perspective offers a deeper understanding of how physical activity can affect positive changes in the brain’s structure and function, leading to improved mood and cognitive function in individuals with MDD [104]. Furthermore, the benefits of exercise extend to the prevention of MDD. According to a longitudinal study by Harvey et al., regular engagement in physical activity can significantly reduce the risk of developing depression. This preventative aspect highlights the role of exercise not just as a therapeutic intervention but also as a vital component of mental health maintenance and promotion [105].

#### 7.1.2. Anxiety Disorders

Anxiety disorders, marked by persistent and excessive worry, are pervasive across the global population, profoundly affecting daily functioning and quality of life. The research conducted by Anderson and Shivakumar sheds light on the substantial benefits that regular participation in sports has for reducing anxiety symptoms [33]. This study emphasizes how physical exertion, coupled with the social aspects inherent in sports activities, cultivates an environment conducive to fostering a sense of mastery, achievement, and overall well-being. The effectiveness of sports and physical activity in alleviating anxiety symptoms is further supported by the work of Biddle and Asare. Their study found that moderate-intensity exercise can lead to significant reductions in anxiety. This is attributed to the role of exercise in modulating the body’s stress response, ultimately leading to decreased levels of stress hormones, such as cortisol, and an increase in endorphins, which are natural mood lifters [106]. Moreover, the social component of engaging in sports cannot be overlooked in its therapeutic value for anxiety disorders. A study by Raglin and Morgan highlighted that social interaction during exercise can enhance the psychological benefits of physical activity, providing a support system and a sense of belonging, which are essential for individuals dealing with anxiety [107].

Additionally, a study by Ensari et al. explored the role of physical activity in modulating the physiological markers associated with anxiety. They reported that regular physical activity leads to improvements in heart rate variability, a marker for stress and anxiety management, suggesting a direct physiological impact of exercise on reducing anxiety symptoms [108].

#### 7.1.3. Post-Traumatic Stress Disorder

PTSD, a condition triggered by traumatic experiences, can profoundly affect an individual’s daily life and mental well-being. The therapeutic value of sports in treating PTSD has been increasingly recognized in recent research. A significant study by Rosenbaum et al. elucidates the beneficial impacts of physical activity on alleviating the symptoms of PTSD [104]. This research posits that regular involvement in sports offers a structured means to manage stress and anxiety, crucial for individuals coping with PTSD. Further reinforcing the effectiveness of sports therapy for PTSD, a study by Powers et al. found that aerobic exercise, in particular, can significantly reduce PTSD symptoms. Their research indicates that aerobic activities, such as running or swimming, contribute to the reduction in hyperarousal symptoms and improve overall mood, which are key factors in managing PTSD [109]. In addition to aerobic exercise, the role of team sports in managing PTSD has been explored. A study by Caddick et al. highlights how participation in team sports provides social support and a sense of belonging, which are particularly beneficial for those with PTSD [110]. This study pointed out that the camaraderie and collective experience in team sports can foster a sense of identity and purpose, aiding in the recovery process.

Yoga, as a form of exercise, has also been examined for its therapeutic effects on PTSD. A research study by van der Kolk et al. demonstrated that yoga can help in regulating the autonomic nervous system, which is often dysregulated in PTSD [111]. Their findings suggest that yoga, with its emphasis on mindfulness and body awareness, can be an effective tool in reducing PTSD symptoms, particularly those related to anxiety and emotional regulation. Thus, sports therapy, whether through individual aerobic exercises, team sports, or practices like yoga, offers a multifaceted approach to alleviating the symptoms of PTSD. The combination of physical activity, social interaction, and structured routine in sports therapy provides a comprehensive treatment modality for individuals dealing with the aftermath of traumatic experiences.

Regarding the type of exercise, resistance training appears to also have an anxiolytic effect. For instance, there is evidence indicating that resistance training has a substantial effect in reducing anxiety. This effect is not influenced by factors such as sex, age, program length, session duration, frequency, intensity, or the extent to which strength is enhanced. A meta-analysis conducted by Gordon et al. demonstrated that resistance training has a substantial positive impact on anxiety symptoms in both individuals who are well and those with a physical or mental ailment [112]. Their results indicated that resistance training effectively alleviates the symptoms of despair and anxiety in patients, not only in healthy people but also in those diagnosed with fibromyalgia. Resistance training is deemed effective in enhancing the mental well-being of fibromyalgia sufferers, hence diminishing their symptoms of despair and anxiety [113]. It is important to mention that an increasing amount of research has found that resistance exercise has anxiety-reducing effects in humans, both after individual exercise sessions and with long-term training [114,115]. In this regard, research findings indicate that engaging in resistance training at a low-to-moderate intensity, specifically below 70% of one’s maximum weightlifting capacity, consistently and significantly reduces anxiety levels. Crucially, the ability to reduce anxiety has been shown in various demographics and measured outcomes. These findings offer validation for incorporating resistance exercise into the clinical treatment of anxiety [116]. Nevertheless, the available data may be limited but indicates that exercise therapies may have the potential to be effective, feasible, and safe non-pharmacological treatments for anxiety and subthreshold anxiety disorders in middle-aged and older individuals [116].

#### 7.1.4. Attention Deficit Hyperactivity Disorder

ADHD is a neurodevelopmental disorder characterized by a persistent pattern of inattention, hyperactivity, and impulsivity. Sports and physical activities have been increasingly recognized for their potential to improve the cognitive and behavioral aspects associated with ADHD. The structured nature of sports activities not only offers an outlet for excess energy but also aids in enhancing concentration, discipline, and social skills. A landmark study by Gapin and Etnier underscored the positive impact of physical activity on the cognitive functioning of individuals with ADHD [117,118]. This study suggested that regular engagement in sports could significantly boost aspects of cognitive function such as memory, attention, and executive control, which are often areas of challenge for those with ADHD. Further supporting this, a study by Verret et al. examined the effects of regular physical activity on children with ADHD [119]. Thus, these findings indicated improvements in attention span and a reduction in disruptive behaviors, highlighting the role of physical exercise in managing ADHD symptoms effectively [120].

In addition, a research study by Smith et al. explored the benefits of team sports for children with ADHD [5]. This study found that participating in team sports not only improved physical health but also had positive effects on social skills and self-esteem, which are crucial for the overall development of children with ADHD. Moreover, the potential of specific sports activities, such as martial arts and yoga, to enhance self-control and mindfulness in individuals with ADHD has been explored. A study by Lakes and Hoyt demonstrated that martial arts training led to improvements in behavior control, attention, and resistance to distractions in children with ADHD [121].

#### 7.1.5. Bipolar Disorder

Bipolar disorder is a complex psychiatric condition characterized by drastic mood swings, encompassing manic highs and depressive lows. Growing evidence suggests that physical activity, including sports, plays a beneficial role in managing this condition. Aerobic exercise is known for its mood-stabilizing effects. Research indicates that activities like running or swimming can positively affect mood regulation in bipolar disorder. These exercises are believed to influence neurotransmitters like serotonin and dopamine, which are crucial in mood regulation [122]. Also, mindfulness and yoga practices have shown promise in managing the symptoms of bipolar disorder, particularly during manic episodes. These practices can aid in stress reduction and enhance emotional regulation [123]. In addition, engaging in regular physical activity has cognitive benefits, which are crucial considering the cognitive dysfunction often observed in bipolar disorder. Exercise can improve cognitive functions like memory, attention, and executive control [124]. Furthermore, participation in physical activities can have social and psychological benefits for individuals with bipolar disorder. Not only can it help improve self-esteem and social skills but also depressive symptoms, which are often challenged in bipolar disorder [125,126].

## 8. Mindfulness in Motion: Incorporating Mindfulness Principles in Sports for Enhanced Well-Being

Mindfulness is a meditation technique, based on vipassana meditation, which was developed by Jon Kabat-Zinn, an American doctor, for clinical purposes. Kabat-Zinn conceptualized this technique as “intentionally paying attention to the present moment”. Its inclusion in sports began with a group of young university rowers trained by Kabat-Zinn in the USA in 1984, who achieved a significant increase in performance improvement after training in this technique [127,128,129,130,131]. Nowadays, there are several ways to include this technique in sports training with different objectives. The main programs used are Mindful Sport Performance Enhancement (MSPE) [132], and the Mindfulness-Acceptance-Commitment (MAC) program [133]. These two programs have been implemented over the last few years and have shown their usefulness in multiple sports modalities. These models, among others used in intervention with athletes, have as a common basis that the athlete must fully accept the experience of conscious contemplation and letting internal events pass without the person trying to control them [134]. It is not that external and internal events are reduced or punished, as in other cognitive–behavioral interventions, but that the person will reduce their emotional reactivity and avoidance behaviors, favoring openness and cognitive flexibility [135].

For the athlete to achieve this attitude of mindfulness, Kabat-Zinn identified a number of domains that need to be learned: non-judgement, patience, beginner’s mind, not seeking to do, trust, letting go/giving in, and acceptance. All of these are acquired with practice and improved with continued training [130,136]. The MSPE program trains present-moment acceptance skills. It usually works in four sessions lasting approximately 3 h [137]. The MAC program combines mindfulness-based cognitive therapy with behavioral modifications and acceptance and commitment therapy. It is usually scheduled in 12 sessions lasting approximately 60 min [127]. These programs require mental training, as well as learning to manage the emotions of the athletes, which will have a positive impact on their psychological well-being and improve their performance in their sporting activity [127]. In this line, previous studies showed that sports performance improved in participants who trained in these programs versus the control group [133,134]. Studies assessing other factors related to coping with competition such as anxiety were able to show that participants reduced their anxiety levels by 2.5%. Assessing personality traits, previous studies suggest that certain personality traits may play an important role in reducing stress and coping strategies in the face of stressful events [134,135].

In the same line, a determinant element in athletes is the so-called burnout, which occurs with a decrease in performance and emotional fatigue [7,137]. Mindfulness-based techniques can help to mitigate this syndrome, although they should be used as a complement to other therapies and interventions [138,139]. Several studies that studied the physiological variables of athletes in competition showed a significant reduction in cortisol levels in saliva, presenting 50% less than before training in mindfulness-based techniques [140,141]. However, in other physiological variables such as heart rate and respiration, no differences were found pre-post. Mindfulness can also be used as a technique for rehabilitation after a sports injury. When an athlete is injured, it is not only the injury itself that has to be considered but also their thoughts of anger, sadness, fear, and low self-esteem [142]. These thoughts and emotions can hinder recovery [135,143]. Previous studies in this line showed that the combination of cognitive behavioral psychological therapy with mindfulness-based techniques helps the athlete to adhere to their treatment, as well as improving the psychological symptomatology associated with the injury [144,145]. All of this has an impact on their recovery, maintaining a positive attitude and helping the person to focus on accepting the moment [131,144]. This technique has shown its benefits in the field of sport as well as in other areas of intervention. Self-regulation, self-awareness, and acceptance are elements that are developed with the practice of mindfulness and provide the athlete with the necessary tools to improve their performance in the practice of physical activity. Undoubtedly, this improves the well-being of athletes, so this technique should be considered an important part of personal and professional development within the sporting context.

## 9. Social Dynamics in Sports: The Psychological Impacts of Teamwork and Camaraderie

Among the human species, there appears to be an inherent inclination towards identification and affiliation with fellow individuals, indicating a fundamental social nature. However, it is worth noting that the intensity of this urge may exhibit variability. The phenomenon of interpersonal communication varies among individuals. Group dynamics has been acknowledged as a significant topic within the discipline of social psychology since its inception. The concept of group dynamics has been employed in two primary ways. Initially, it has been employed to portray the dynamism and evolving characteristics of collectives. Furthermore, sociology is widely regarded as the academic discipline that primarily examines the patterns of behavior exhibited by collectives [145]. A number of social scientists have engaged in extensive discourse over the significance of the psychological advantages that arise as a result of group dynamics [146]. Affiliation and social identification with others and the incorporation of individuals who are engaged in activities within a certain social identity [147].The term “framework” refers to a conceptual structure or model that provides a basis for understanding, organizing, and analyzing a certain subject. Generally speaking, these aspects show that, for human well-being, emotions play a significant role. The concept of identification with esteemed social groups and collectives contributes to the advancement of a social networking platform that offers psychological services [147]. An individual who possesses good mental health is characterized by reduced occurrences of conditions such as depression, anxiety, and loneliness, among others [148]. In this regard, Andersen et al. pointed out in a recent review that there exists a body of consistent research indicating that engagement in team sports is linked to enhanced social and psychological well-being, irrespective of the specific team sport, age, or presence of somatic or mental health issues [149]. Their results suggested that team sports may be more effective in promoting health and facilitating exercise engagement and adherence compared to individual sports. Nevertheless, it is imperative to use caution while employing team sports for the aim of promoting health due to the inherent competitive tendency associated with such activities [150]. Nevertheless, Guddal et al. revealed that the level of physical activity (PA) matters, and a high level of PA improves multiple aspects of mental health, particularly among adolescents attending senior high school. Participation in team sports has the potential to have favorable effects on mental well-being, thus warranting promotion and encouragement [151]. Related to this, the presence of a coach who is supportive and focused on mastering appears to have positive effects on the psychosocial development of young athletes. On the other hand, athletes who face simultaneous pressure to perform from both their coaches and parents may have fewer psychological benefits from participating in sports [152].

With the underlying premise that there exists a correlation between social identity and psychological health and well-being, Graupensperger et al. tried to gain insights into the manner in which social ties and group structure within college club sport teams are associated with students’ perceptions of social identification [153]. In this study, a total of 852 student athletes were selected as participants, specifically chosen from 35 entire same-sex collegiate club sport teams. There was a positive association observed between outdegree centrality, indegree centrality, team density, and the strength of athletes’ social affiliation with their sport team [153]. Also, upon comprehensive examination of the research outcomes, it became apparent that there exists substantiation supporting the notion that sharing physical activities (unstructured free play) can facilitate the development of cooperation, task coherence, and leadership skills and foster an inclination towards friendship approach goals [154]. Additionally, the impact of objectives on performance can be observed through their ability to direct attention towards the work at hand, foster perseverance, and enhance both the effort and adoption of novel performance tactics [155]. Also, the significance of game playing is underscored by the emphasis placed on teamwork, cooperative play, and good sports behavior [156]. However, Senecal et al. specify that when analyzing the challenges encountered by contact sport athletes in their career transitions, it is imperative to explore the distinctive impact that the social aspects of camaraderie and solidarity have had on these athletes throughout their professional journeys [157]. Therefore, the incapacity to establish substantial connections post-sport may have a substantial influence on the challenges encountered by athletes during their job transitions. These challenges encompass psychological, motivational, emotional, and social conflicts, as emphasized in the examination of this particular demographic. In this regard, the research conducted by Miller and Kerr illustrates the prevalence of a triangle of tension experienced by student athletes, encompassing their athletic demands, academic obligations, and social needs. The findings of this study indicate that student athletes consistently prioritize their athletic and academic obligations, often sacrificing social engagements [158]. Moreover, when faced with a conflict between athletic and academic responsibilities, student athletes tend to choose their athletic commitments above their academic commitments. In fact, as evidenced, it became evident that individuals are unable to effectively reproduce the emotions of unity, integrity, and encouragement that were encountered within the context of being teammates in physical sports [159]. Without intending to exaggerate the magnitude of the loss, the absence of a daily sense of emotional proximity to those in one’s social circle can potentially result in a fairly distressing experience [160]. When considering the conjunction of cultural norms around masculinity with respect to male contact sports and the societal expectation of maintaining a stoic disposition towards emotions and interpersonal connections, it becomes evident that a significant psychosocial dilemma may arise [161]. In a study conducted by Weiss and Smith et al., it was discovered that adolescent tennis players between the ages of 14 and 18 had higher ratings for loyalty, closeness, commonality, and conflict compared to younger players aged 10 to 13. Conversely, the younger players rated friendship and joyful play higher in comparison. Female participants exhibited higher ratings in terms of self-esteem, support, loyalty, intimacy, and commonality, whereas male participants assigned higher ratings to conflict [162].

Regarding motivation, it refers to the underlying causes that serve to motivate an individual’s conduct, guiding them towards a specific course of action and facilitating coordination as a result. Individuals exhibit variations not just in their aptitude for a certain task but also in their inclination or motivation to engage in that task. Understanding the underlying motive that drives behavior in sport participation is of significant importance. It is crucial to ascertain the catalyst that initiates such activity and identify the factors that may potentially modify it [85]. Moradi and colleagues studied 265 athletes from various disciplines, including men and women. Their findings indicated a statistically significant disparity in the factors driving motivation for sport involvement among athletes belonging to team sports compared to those participating in individual disciplines, as well as between male and female athletes. Female athletes predominantly engaged in sports for the purposes of fostering teamwork, deriving enjoyment, and enhancing physical fitness [163]. Conversely, male athletes typically participated in sports due to situational factors, the enjoyment derived from the activity, and the promotion of teamwork. Based on the findings presented, it is recommended that coaches and sports management consider these aspects in order to optimize participation rates and ensure ongoing engagement in physical activity. Additionally, it is advisable to undertake a research study to examine the various aspects that influence the motivation of athletes to participate in different sports disciplines, including athletic tournaments [163].

**Figure 2:**
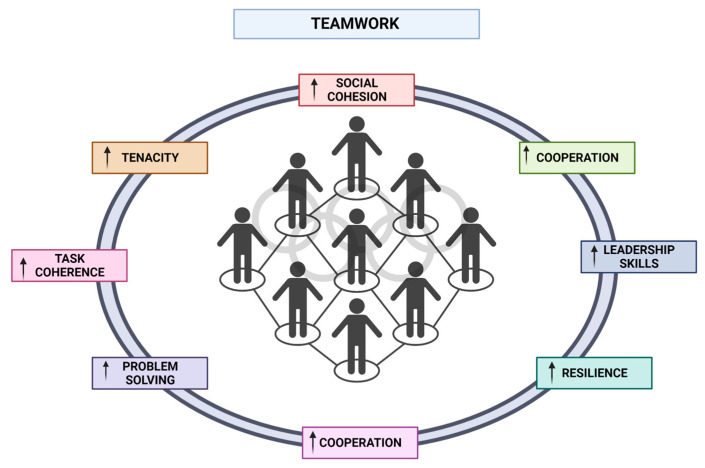
Psychological effects of team building in sports.

In summary, studies indicate that the presence of group goals and the promotion of teamwork have a substantial impact on social cohesion (Figure 2). Furthermore, it was observed that communication plays a mediating role in the relationship between teamwork and task cohesion [164]. For example, ultimate frisbee is distinguished by the presence of communication among all participants, regardless of their affiliation with either the same or opposing team. In addition, individual sports also offer various health benefits, including contact sports or fencing [6]. It has been found to have positive effects on various aspects of physical fitness, including cardiovascular fitness, muscular strength, and endurance, as well as general body coordination. Furthermore, it elicits cognitive faculties such as problem-solving, critical thinking, and strategic planning. From an educational standpoint, the concepts of self-control, collaboration, and sportsmanship [165]. Sports programs offer a secure setting for adolescent individuals to effectively cope with stress and regulate their emotional responses [166]. Additionally, it fosters personal growth and character development through the cultivation of qualities such as resilience, tenacity, and determination. However, future directions should elucidate the fundamental strategies employed by sport team leaders to optimize team performance and promote the overall welfare of their athletes. Findings indicate that the establishment and reinforcement of a collective identity within a team facilitated the development of a psychologically secure atmosphere. This, in turn, facilitated the emergence of both a team-oriented approach and an individual-oriented approach, with particular emphasis on the role of informal athlete leaders [167].

## 10. Environmental Influences: How Settings, like Gyms or Nature, Impact Psychological Outcomes

The consensus among scholars is that engaging in PA for an extended period of time has a positive impact on both physical and mental well-being throughout one’s lifetime [168]. The influence of the natural environment on an individual’s level of physical activity can be attributed to the provision of appropriate circumstances for engaging in specific types of activities. Natural environments offer convenient and easily accessible venues for physical activity, enticing individuals to engage in outdoor activities due to the distinctive experiences they provide, in contrast to physical activity opportunities in urban or artificial settings. Empirical evidence suggests that individuals residing in close proximity to natural environments exhibit elevated levels of physical activity. Additionally, individuals with elevated levels of PA exhibit a tendency to engage in more frequent and extended visits to natural environments [169]. Generally, the affordances present in natural situations differ from those found in constructed or urban contexts. The recognition of these affordances rooted in nature entails a range of perceptual and behavioral flexibility that attracts individuals to establish and maintain a deep connection with the natural surroundings, including physical, psychological, and/or emotional dimensions [170].

In this regard, it is known that engaging in physical activity within natural settings has been found to have a good impact on cognitive function, emotional well-being, and the mitigation of psychological stress. Nevertheless, there is still a lack of clarity regarding whether these advantages are influenced by a gradient effect, in which case, it is uncertain whether natural traits of a bigger magnitude result in more pronounced cognitive and psychological benefits. Wade et al. discovered that engaging in indoor exercise for a short duration may enhance sustained attention accuracy to a greater extent compared to engaging in exercise in a park setting [171]. Nevertheless, this study did not yield any empirical data supporting the notion of an environmental impact on working memory. It is noteworthy that engaging in physical activity within a park setting has been demonstrated to yield enhancements in overall well-being at the state level. Also, the findings of this study do not provide sufficient evidence to support the idea of a ‘nature gradient’ that was investigated [171]. Lahart and colleagues were more specific and pointed out that, when comparing indoor exercise to acute episodes of outdoor green exercise, it has been seen that the latter has a positive impact on affective valence and enjoyment. However, there is no significant effect on emotion, perceived exertion, exercise intensity, or biological markers. No additional statistically significant differences were detected, save for a greater preference for outdoor green activity compared to virtual green exercise. A significant propensity for bias was seen across the trials, indicating an overall substandard quality of evidence. In summary, the available evidence is limited in its support for the notion that engaging in green exercise provides superior advantages compared to engaging in exercise without exposure to natural environments [172].

Compared to this, focusing on individuals between the ages of 18 and 21 who are classified as obese reaffirmed prior research indicating that engaging in physical exercise in outdoor surroundings is more effective in alleviating stress and enhancing attentional capacity compared to indoor activities [173]. These findings indicate that the participants’ mood states and environmental perceptions may be impacted by the varying walking situations, including diverse settings and speeds [173]. The results of this study can be applied to the development and arrangement of urban green areas with the aim of encouraging physical activity and formulating exercise programs specifically tailored for individuals with obesity [174]. In a study conducted in Finland, researchers conducted a field experiment to investigate the psychological and physiological impacts of visiting urban natural environments. This study revealed that when contrasted to a densely populated city center, both an urban park and urban woodland had a beneficial influence on stress recovery [175]. The potential substitution of outdoor exercise with physical activity in a gym setting raises the need to examine and compare the effects of natural environments and gym sites on physical activity programs, as this could have significant ramifications. Additionally, a research conducted in the United Kingdom examined the impact of engaging in treadmill exercise while being exposed to nature photos projected on a wall. The findings suggested that including green exercise, which involves exposure to natural environments, can lead to greater improvements in self-esteem compared to exercise alone [176]. Also, in a study conducted by Bowler et al., the effects of exercise in natural contexts were compared to “synthetic” environments. For the sake of this study, synthetic environments were defined as indoor settings and outdoor-built environments that lacked greenery. The researchers concluded that the natural environment has a significant impact on emotions such as anger, exhaustion, serenity, and sadness, with a slightly beneficial influence on energy levels [177].

Regarding gender, studies have indicated that there may be a connection between gender and the impact of the ambient setting of physical activity on psychological effects. According to Bengoechea’s study [178], it was observed that women tend to report lower levels of felt exertion while outdoor running compared to their real heart rate, whereas men tend to report higher levels of perceived exertion than expected. According to the studies conducted by Barton and Pretty [176,179], it was observed that men exhibited slightly greater enhancements in mood as a result of engaging in green exercise compared to women. Moreover, there is evidence to suggest that there are gender differences in behavior and attitudes towards natural surroundings. According to Puett et al. [180], there is a higher likelihood of men participating in physical activities outside. Conversely, Zelezny et al. discovered that women are more inclined to engage in pro-environmental behaviors and may possess a stronger sense of connection to the natural environment [181]. The potential link between individuals’ connection with nature and their health has been explored by Cervinka et al. [182]. The results of their study suggest that women might derive more significant psychological advantages from engaging in green exercise compared to males [182].

**Figure 3:**
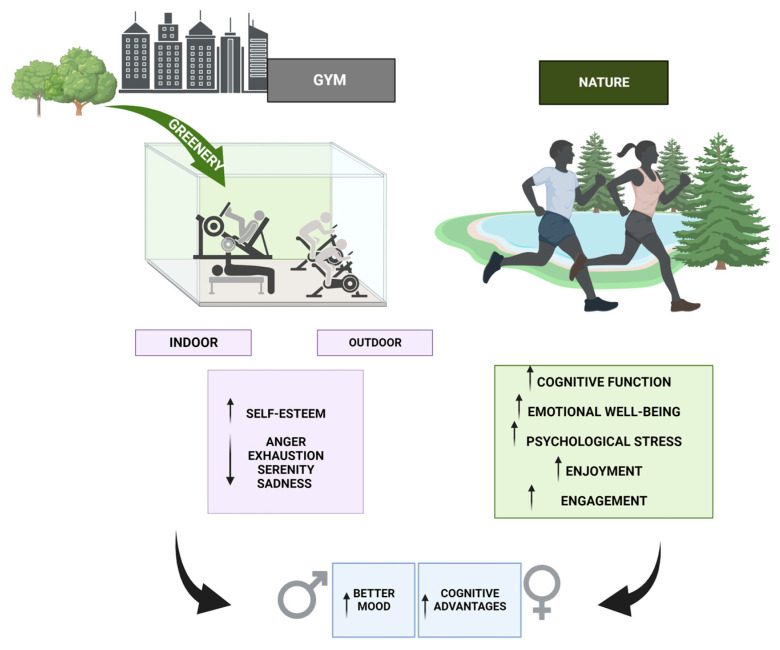
Effects of training in contact with nature or in environments with reference to nature.

In summary, the natural environment is beneficial for anxiety, anger/hostility, vitality, affect, and positive engagement (Figure 3). Outdoor physical activity in natural environments is more beneficial for a variety of psychological outcomes than urban environments. The ramifications of these findings are significant in the context of urban planning and the implementation of green social prescribing for mental health.

## 11. The Double-Edged Sword: Recognizing and Addressing Exercise Addiction

Exercise addiction represents a significant, yet often overlooked, concern in the context of physical activity and sports psychology. While exercise is universally promoted for its health benefits, when it becomes compulsive and excessive, it can lead to negative consequences, both physically and mentally [183]. This condition, characterized by an obsessive compulsion to exercise and the prioritization of exercise above other important activities, raises important questions about the balance between healthy and harmful exercise behaviors [184]. The complexity of exercise addiction lies in its dual nature. On the one hand, exercise is a healthy behavior; on the other, when it becomes excessive, it can lead to physical injuries, psychological distress, and social isolation [185,186]. The prevalence of exercise addiction is not fully understood, but it is believed to be higher among specific populations, such as athletes and regular gym-goers, who are more exposed to competitive and high-intensity training environments [187].

Identifying exercise addiction can be challenging because it often masquerades as a healthy commitment to fitness. Symptoms may include withdrawal effects in the absence of exercise, an increase in the amount of exercise needed to feel satisfied (tolerance), a lack of control over exercise habits, and a noticeable reduction in other life activities [188]. These symptoms, together with negative consequences such as injuries, fatigue, and social withdrawal, are key indicators of a potential problem [189]. The risk factors for developing exercise addiction include personality traits, such as perfectionism, high levels of neuroticism, and a predisposition to addictive behaviors. Moreover, cultural and societal pressures to maintain a certain physique can exacerbate tendencies towards excessive exercise, especially in contexts where physical appearance is highly valued [190].

Psychologically, exercise addiction is often interlinked with various mental health issues, such as anxiety, depression, and, notably, eating disorders. Individuals with exercise addiction may use excessive physical activity as a means to cope with or control these psychological challenges, creating a vicious cycle where mental health issues perpetuate the addiction, and the addiction exacerbates the mental health issues [185,186,189]. This complex interplay suggests that exercise addiction is not merely a physical issue but is deeply rooted in psychological well-being. Moreover, exercise addiction has been associated with certain personality traits, including perfectionism, high self-standards, and a tendency towards obsessive-compulsive behaviors [191,192]. These traits can predispose individuals to become overly involved in exercise routines, blurring the line between healthy engagement and compulsive behavior. Additionally, the role of self-identity in exercise addiction is significant; for many, their self-worth and identity become tightly intertwined with their physical fitness and exercise achievements [183].

Physiologically, the implications of exercise addiction are profound and multifaceted. Chronic over-exercising can lead to a range of physical health problems, including overuse injuries, hormonal imbalances, and extreme fatigue [193]. The continuous strain on the body without adequate rest and recovery can escalate into more serious conditions, such as stress fractures, muscle strains, and cardiovascular issues [194]. From an endocrine perspective, excessive exercise can disrupt the delicate balance of hormones in the body, leading to the development of certain conditions, such as adrenal fatigue and reproductive health issues. In women, for example, exercise addiction can lead to amenorrhea and osteoporosis, a condition often referred to as the female athlete triad [195]. In men, prolonged over-exercise can result in decreased testosterone levels and reduced bone density [196,197].

Socially, exercise addiction often leads to significant disruptions in personal and professional relationships. Individuals with exercise addiction tend to prioritize their exercise regimen over family time, work commitments, and social interactions, which can result in strained relationships and social isolation [198,199,200]. The compulsive nature of this addiction can make it difficult for individuals to maintain a balanced lifestyle, leading to conflicts with loved ones and colleagues who may feel neglected or secondary to the exercise routine. The role of social media and technology in perpetuating exercise addiction is increasingly recognized. Platforms like Instagram and Facebook are inundated with fitness-related content, often promoting unrealistic body standards and glorifying over-exercise. This constant exposure can fuel exercise addiction, especially among younger populations who are more susceptible to social media influences [201,202]. The interactive nature of these platforms, where likes and comments serve as validation, can exacerbate the compulsive need to exercise and share fitness achievements.

Culturally, exercise addiction varies across societies and is influenced by cultural norms related to body image, health, and fitness. In Western cultures, where there is a significant emphasis on individualism and appearance, the prevalence of exercise addiction is notably higher [203,204]. This cultural dimension highlights the importance of considering societal values and beliefs when addressing exercise addiction. Moreover, the fitness industry itself, with its focus on high-intensity workouts and transformation challenges, can contribute to the normalization of excessive exercise. Fitness influencers and trainers often promote rigorous training schedules and extreme fitness goals, which, while motivating for some, can push vulnerable individuals towards addictive behaviors [205,206].

Addressing exercise addiction necessitates a comprehensive approach that includes identifying and treating any underlying psychological disorders. Psychological interventions, such as cognitive behavioral therapy (CBT), have been proven to be effective in treating exercise addiction by aiding individuals in developing healthier coping strategies and altering their exercise-related thought and behavior patterns [207]. Additionally, mindfulness techniques and acceptance and commitment therapy (ACT) can be beneficial, as they assist individuals in increasing awareness and accepting their thoughts and feelings without engaging in compulsive actions [208]. Education about the risks of exercise addiction and the promotion of healthy exercise habits are key to prevention and treatment. Fitness professionals and coaches play a critical role in educating clients about the importance of rest and recovery and in encouraging a balanced approach to exercise [209,210]. This includes setting healthy limits, recognizing the signs of fatigue and overtraining, and avoiding the promotion of unrealistic body ideals or extreme exercise routines.

Preventative measures also involve raising awareness about the signs of exercise addiction among fitness professionals, coaches, and the general exercising population. Early identification of risky behaviors can prevent the escalation to full-blown addiction [211]. Furthermore, support from family and friends is crucial; they can help identify concerning changes in exercise patterns and provide emotional support during treatment and recovery [208]. Finally, collaboration between mental health and sports professionals can enhance treatment outcomes, allowing for a more holistic approach that addresses both the physical and psychological aspects of exercise addiction [207]. This multidisciplinary approach is essential for effectively treating exercise addiction and promoting a healthy and balanced lifestyle.

## 12. Cultural Lenses: Understanding Sports and Psychology from Diverse Cultural Standpoints

In an increasingly interconnected and diverse world, we delve into the multifaceted relationship between culture, sports, and psychology, shedding light on the role culture plays in shaping our perceptions, behaviors, and attitudes in these domains. In order to understand how cultural diversity permeates and influences sports and psychology, a nuanced examination of standpoints that shape these fields is required. As culture can be defined as a set of attitudes, values, beliefs, and behaviors shared by a group of people, but different for each individual, communicated from one generation to the next [212], these elements are expressed through art, rituals, customs, and institutions.

Sport is a cultural product that embodies and expresses the social, historical, and individual dimensions of a community, but it also serves as a powerful platform for the expression of identity, the reinforcement of social bonds, and the transmission of cultural values across generations. Spaaij et al. refer to Vermeulen and Verweel as researchers who examined the social impact of sport through the lenses of sociocultural diversity, also making reference to the processes of social inclusion and exclusion [213]. Looking at this subject from a different perspective, it introduces a compelling metaphor in which sport serves as a symbolic dialogue, portraying a dramatic representation of our identity and our aspirations [213].

Psychology in the context of culture and sport reflects how individuals’ mental processes and behaviors, as well as their emotional and social experiences in sports, are influenced by motivation, performance enhancement, mental resilience, goal setting, stress management, and team dynamics within the realm of sports and exercise [214]. These psychological facets are universal and deeply embedded in the diverse cultural values and expectations that shape the athletes’ experiences. Moreover, cultural elements can significantly impact athletes’ psychological states, attitudes, and reactions to various aspects of sports, from competition to victory and defeat. Successful athletes and teams can become cultural icons, and the psychological strategies employed in sports can extend beyond the playing field, impacting the broader aspects of our society [215].

This triad represents the intricate interplay of mental processes, social norms, values, and practices within the context of sports and physical activities. This symbiotic relationship encompasses the psychological aspects of human performance in sports, exercise, and physical endeavors, which are profoundly influenced by the cultural milieu in which they occur [216]. If we look at the significance of self-identity, it is more closely linked to individualistic cultures as opposed to collectivistic ones. In countries with an individualistic orientation, such as many Western societies, the importance of personal identity within close-knit working relationships, like those found in team sports, tends to be more pronounced than in collectivistic cultures [217].

This heightened emphasis on identity in individualistic nations can be attributed to the distinct separation of work and leisure. Individualistic cultures encourage the development of a unique sense of self and autonomy, drawing clear boundaries between one’s personal identity and that of others. These cultures prioritize the individual’s needs, desires, and aspirations over group or collective interests [217]. Conversely, collectivistic cultures foster an environment where the needs, wishes, and desires of in-groups take precedence over those of individuals. Values such as harmony, cooperation, cohesion, and conformity are highly esteemed within these cultures. An illustrative example can be found in the difference between individualistic individuals, like Americans, who actively engage in self-enhancement and self-identity development, and collectivistic individuals, such as the Japanese, who are more inclined toward self-criticism and the cultivation of group identities [218].

Different cultures exhibit varying degrees of passion and enthusiasm for sports, often elevating specific sports to the status of national or regional pastimes. For example, soccer (or football) is not merely a sport but a cultural phenomenon. It unites communities, transcending boundaries and forging a sense of shared identity. In contrast, cricket reigns in South Asia, where it serves as a source of national pride and represents the continuation and unity of the Indian identity, as well as being an ideal tool for nation building [219]. Sport also helps shape New Zealand’s national identity, and the haka provides a unique sense of distinction in relation to the rest of the world [220]. Haka is more than just a ritual dance but represents a cultural philosophy that embodies values such as integrity, passion, solidarity, discipline, and respect [220]. Playing Haka can have a profound impact on one’s psychological well-being, promoting a positive sense of identity and connection to one’s cultural roots. It also serves as psychological mechanisms for cultural transmission, reinforcing the shared identity and historical narratives within a community.

Sumo wrestling in Japan is another example, deeply rooted in tradition, rituals, and symbolism, reflecting the unique cultural values of the country [221]. Sumo is a clear reference to the Shinto religion and to the dances that, in ancient times, were practiced in temples by wrestlers. Accepting the condition of transforming their own body in order to achieve victory and notoriety means that they have understood the philosophy of Sumo, are to this legendary sport, and are ready to put its teachings into practice. Sumo wrestlers engage in arduous training routines and strictly adhere to a code of conduct that highlights the significance of commitment, resilience, and self-discipline [221]. These principles are deeply embedded in the fabric of sumo, mirroring the overarching cultural virtues cherished by Japanese society.

Taking the cross-cultural perspective into account, it becomes obvious that the situational context may differ severely in different countries or regions. In some cultures, for example, pride after a victory over a strong sportive rival is demonstrated without inhibition, while athletes in other countries suppress such a feeling as a sign of respect for the defeated opponent. Duda and Allison suggested that the existing cultural variability in motor development, physical as well as sport performance, and exercise involvement should be further analyzed by sport psychologists at a conceptual level to identify possible psychological factors (such as motivation, expectations, perceived ability, etc.) influencing cultural differences [222]. At the same time, its social, cultural, and functional aspects vary according to the cultural background. For example, at the Olympic Games, various athletes from different countries, races, and cultures play on the same field under the same rules with a fair play spirit. Therefore, according to Si and Lee [223], “a well-designed cross-cultural study can help us to know and understand more social, psychological aspects which influence sport performance in different races”.

We have seen that through the lens of culture, we gain insights into the profound impact of cultural values, norms, and traditions on the perception and practice of sports and psychology. Whether examining the role of sports in uniting communities and forging identities or delving into the cultural nuances that influence athletes’ psychological experiences, the intricate relationship between culture and these fields becomes evident. Moreover, the importance of cultural competence in sports psychology cannot be overstated, as it promotes inclusivity and effective support for athletes from various backgrounds. As we move forward, exploring the cultural dimensions of sports and psychology opens up exciting avenues for research, intervention, and progress in these domains, fostering a richer, more inclusive, and empathetic understanding of the human experience in the context of sports and psychology.

## 13. Gender Perspectives: The Varying Impacts and Perspectives of Sports on Psychological Health

The power of sports as a tool for promoting positive psychological health has a long and storied history, dating back to ancient times. With their deep appreciation for the connection between physical and mental well-being, the ancient Greeks embodied this concept through the famous Hippocratic ideal, “Healthy body in a healthy mind”. Through the ages, this idea has persisted, evolving into a fundamental principle of sports and exercise psychology, underlining the profound impact of sports on psychological health and well-being. Within the mosaic of diversity, gender plays a pivotal role as one of the fundamental aspects of human identity and culture. In this context, sport and exercise psychology research draws attention not only to the inclusion of gender issues but also to the gendered cultural confines of elite sports. On the other hand, many sports’ academics use “sex” and “gender” interchangeably, although the distinction between both terms assumes significance through the delineation of intrinsic biological attributes versus culturally constructed roles and identities. Sex is used to label the dichotomous distinction between females and males based on physiological characteristics that are genetically determined, whereas gender is used to label the psychological, cultural, and social dimensions of masculinity and femininity. Sex is a biological category predicated upon the differentiation of reproductive structures, chromosomal composition, and hormones. In contrast, gender, operating within the sociocultural milieu, extends beyond the purview of biology to encompass an intricate interplay of identity, expression, and societal roles. Recognizing the non-binary nature of gender, where individuals may identify beyond the conventional male/female binary, underscores the necessity of transcending simplistic categorizations and acknowledging the complexity inherent in both the biological and sociocultural dimensions of sex and gender. Femininity and masculinity refer to an integral expression of the gender spectrum as identity, values, behavior, and appearance experiences. The idea that sex and gender in sports are treated as separate entities, each having its own characteristics and significance, is a key concept that has a meaningful relationship. This viewpoint allows for a nuanced understanding of how biological and sociocultural factors interact in shaping the experiences and performances of individuals in sport [224].

Gender differences are traditionally depicted in binary terms, distinguishing between femininity and masculinity. However, this binary categorization oversimplifies the complexity of gender experiences, as both masculinity and femininity can manifest in diverse ways among individuals. These variations are often influenced by factors such as ethnicity, socioeconomic background, age, generational cohort, and sexual orientation [225]. The perpetuation of myths and stereotypes by the media has had a detrimental impact on women’s participation in sports [226]. Gender stereotypes pertain to societal expectations regarding the suitable traits and behaviors for individuals based on their gender, and these stereotypes can manifest differently within specific contexts, including the presence of gender stereotypes in sport [9]. The recurrent conventional gender roles portray individuals in stereotypical ways, as, for example, women are depicted in more ornamental roles, emphasizing their visual and aesthetic attributes, while showcasing men as authoritative figures in positions of power [227]. Moreover, women are portrayed as passive and often in traditionally feminine poses, creating images that reinforce gender stereotypes, and although they are distorted and false, they deeply influence our understanding of ourselves and the society we live in [228].

The field of sports has historically been considered a masculine realm, and women were excluded, with gendered barriers often obstructing their path. These barriers, shaped by deeply ingrained stereotypes and historical norms, have created significant challenges for women athletes striving to break free from the constraints of traditional gender roles. However, despite these obstacles, women in sports have persevered, pushing boundaries, shattering stereotypes, and proving their prowess as athletes. Their determination and resilience serve as evidence of the transformative power of women’s inclusion in the world of sports, highlighting the need for continued efforts to ensure equal opportunities for all [229]. The psychological benefits of sports involvement can vary significantly based on an individual’s gender and the sociocultural context in which they engage in sports. Andersen, Ottesen, and Thing, suggested that participation in a team sport is associated with improved social and psychological health independent of the type of team sport, age, somatic, or mental health problems [150]. Team sports may offer greater effectiveness in promoting health and sustaining exercise engagement compared to individual sports. Nonetheless, when harnessing team sports for health-related purposes, it is essential to be cautious concerning their intrinsically competitive characteristics [150].

Men and women often experience sports differently due to a complex interplay of sociocultural factors, and these differing perspectives can influence psychological health outcomes. Women often have a tendency to underestimate their physical capabilities more so than men [150]. Consequently, cultural factors have historically imposed more limitations on women’s full participation in sports compared to men. If it is supposed that disparities in sports abilities between men and women are attributed to physiological differences, English (2020) suggested that what might be seen as a physiological disadvantage in one sport for women could be an advantage in other sports. For instance, weight is an asset for sumo wrestlers but a disadvantage for football players. Conversely, in sports emphasizing traits like flexibility, balance, strength, timing, and smaller body size, women could excel. Furthermore, these sports characteristics, such as balance and strength, can be just as appealing to spectators as those traditionally associated with men, like strength and speed. However, most contemporary sports have been tailored to suit men’s physiology, reflecting their historical dominance in the field of sports [230].

In the realm of sports, women often find themselves reaping a host of psychological benefits, including increased self-confidence, improved emotional regulation, and stress reduction. Engaging in sports can serve as a powerful tool to empower women, enabling them to challenge preconceived gender stereotypes and cultivate a positive self-concept. Nevertheless, women may encounter unique hurdles, such as the societal expectation to conform to specific body image standards and the need to confront gender-based discrimination within the sporting arena [231]. On the other hand, men who participate in sports frequently experience heightened self-esteem, enhanced self-efficacy, and a reduced sense of stress. Involvement in traditionally masculine sports often reinforces feelings of competence and accomplishment, contributing to an overall sense of psychological well-being. Nonetheless, the pressure to conform to these predefined roles can sometimes lead to emotional suppression, and there is an elevated risk of sustaining sports-related injuries among male athletes [159].

As we have seen, the field of sport psychology has made significant strides in recognizing and addressing the profound impact of gender on athletes’ experiences and performance. Gender in sports psychology is not just a binary construct; it is a complex interplay of sociocultural factors that influence how individuals engage in sports and physical activities. These factors can give us a holistic understanding of how they can affect athletes’ self-esteem, motivation, stress levels, and overall psychological well-being. In this sense, it is essential to continue challenging stereotypes and promoting inclusivity, ensuring that athletes of all genders have equal opportunities and support to thrive in sports. Ultimately, embracing a diverse and inclusive perspective on gender in sport psychology contributes to the overall growth and well-being of athletes and enhances the richness of our sporting experiences.

## 14. Performance Enhancement and Mental Strategies

The training process of athletes, ranging from beginners to elite competitors, is influenced by biological, psychological, perceptual–cognitive, and social factors [232]. Psychological components can be crucial during training and competition, regardless of the sport [233]. Therefore, psychological interventions are beneficial in enhancing both psychological well-being and athletic performance [234]. These effects are achieved through improvements in cognitive skills, such as motivation, mental concentration, and self-confidence [235,236].

### 14.1. Cognitive Behavioral Therapy in Sports

Cognitive behavioral therapy (CBT) has been effectively implemented in training programs for athletes and sports teams, yielding significant results in enhancing mental skills. These improvements are attained through a comprehensive training approach, encompassing physical, technical, and tactical training [236]. The relevance of psychological interventions in sports is increasingly recognized for their role in enhancing psychological well-being and optimizing athletic performance. Moreover, the training and/or teaching of psychological strategies improves vital psychological skills in sports, such as mental concentration, motivation, arousal levels, etc., which are crucial for athletic performance [237]. High-level athletes and competition participants often undergo numerous and rigorous training sessions, which can lead to potential physical and mental overload and fatigue. Addressing these issues is essential to prevent negative impacts on their health and performance [238,239].

Psychological training encompasses various techniques that facilitate the learning, maintenance, and enhancement of motor and cognitive skills in athletes [240,241]. Among the most effective psychological intervention techniques to optimize and improve athletic performance are mindfulness acceptance and commitment therapy [242,243], stress inoculation therapy, emotional freedom techniques, psychological skills training, and cognitive behavioral therapy (CBT) [244]. Psychological skills, in themselves, do not produce superior athletic performance beyond an athlete’s potential. However, they can aid in achieving a performance level as close as possible to their maximum potential, in conjunction with physical, tactical, and technical training [245]. The implementation of CBT has shown remarkable results in enhancing the mental skills of many athletes and sports teams [234], as well as in emotional control [243,244,245].

In beginner athletes, enhancing cognitive skills is crucial for increasing adherence to sports practice, managing competition stress, and coping with defeats [246,247,248]. These phenomena should be preemptively addressed to avoid associated consequences, such as increased anxiety or concentration difficulties [249,250]. Prior to and during competitions, stress has been linked with motivation and heightened attention resources, provided that the stress level is manageable and combined with problem-focused coping styles [251,252,253].

### 14.2. Visualization Techniques in Sports

Visualization is defined as the representation of an object or phenomenon in its absence [254]. Its use dates back thousands of years and has significantly evolved in recent decades [255]. Visualization involves evaluating an altered state of consciousness, altering biochemistry and brain activity, thereby enhancing healing and performance [256]. Imagery serves cognitive and motivational functions, both generally and specifically [257]. In sports, mental training techniques incorporating imagery are employed to optimize athletic performance [258]. Imagery, which can stem from both reflective and unconscious memories, plays a critical role in enhancing performance in motor tasks [259].

Various studies using the Sport Imagery Questionnaire (SIQ) have investigated the frequency of imagery use among athletes practicing different sports. The SIQ explores two cognitive factors: Specific Cognitive Imagery (execution of movements and techniques) and General Cognitive Imagery (tactics and action plans). The other three factors are Specific Motivational Imagery (winning, achieving successful performances, and reaching goals), General Motivational Arousal Imagery (emotional excitement related to sports competition), and General Motivational Mastery Imagery (emotion control during challenging situations) [260]. The effectiveness of imagery and how athletes use it are influenced by variables such as gender, skill type (open or closed), competitive level (elite vs. non-elite athletes), and types of sports (team sports vs. individual sports; contact vs. non-contact sports) [259]. Studies have found differing uses and capabilities in imagery among male and female athletes, as well as between elite and non-elite players, in various sports [261,262,263,264,265,266,267,268,269,270,271].

### 14.3. Mindfulness-Based Interventions in Sports

Mindfulness-based interventions (MBIs) have been widely applied in competitive sports concerning athletic performance and mental health promotion [242]. Originating from Eastern Zen, mindfulness is defined as a focused, non-judgmental, conscious, and deliberate awareness of our surroundings in the present [272]. It involves being mindful of internal and external thoughts and feelings without judgment [273]. In sports, mindfulness is used to enhance athletes’ acceptance and performance capabilities, effectively managing attention and improving bodily perception [236]. It helps eliminate prior emotional states of worry, avert performance failure under pressure, and adjust athletes’ mental states [273]. Mindfulness is positively associated with improved athletic performance, enhanced mindfulness, related psychological components (e.g., acceptance, flow, and psychological flexibility), and reduced risks of mental health issues (stress, anxiety, depression, and burnout) [274]. Studies have demonstrated the impact of mindfulness sessions on athletes’ performance and anxiety levels in various sports, showing benefits in both elite and amateur athletes [275,276,277,278,279,280,281,282].

## 15. Eating Disorders and Relative Energy Deficiency in Sport (REDs)

Clinically diagnosed mental illnesses, such as eating disorders (EDs), can manifest in athletes, both male and female, linked to the physical and psychological demands of competition. EDs are psychiatric disorders with diagnostic criteria based on psychological, behavioral, and physiological characteristics [283]. Typically, EDs are chronic and have a significant negative impact at both the physiological (neuroendocrine) and psychosocial levels on those affected. These disorders are characterized by a preoccupation with food, body weight, and body image (physical self-concept), leading to behaviors like starvation, fasting, binge eating, purging, and excessive exercise [284]. The propensity to develop an ED depends on sociocultural, demographic, environmental, biological, psychological, and behavioral factors [285]. The Diagnostic and Statistical Manual of Mental Disorders (DSM-V, 2013) [286] classifies EDs into various specific types, including Anorexia Nervosa, Bulimia Nervosa, Binge Eating Disorder, Pica, Rumination Disorder, Avoidant/Restrictive Food Intake Disorder, Other Specified Feeding or Eating Disorder, and Unspecified Feeding or Eating Disorder [285]. Individuals diagnosed with EDs often exhibit other psychiatric disorders, classified as Axis I psychiatric disorders, which include depression, anxiety, body dysmorphic disorder, or substance dependence, and Axis II personality disorders such as borderline personality disorder frequently observed in the ED population. The characteristics of these disorders increase the complexity of treatment and require additional counseling skills [283].

### 15.1. Eating Disorders and Sport

Numerous risk factors have been discussed for the development of EDs in sports, including general and gender-specific risk factors, predisposing factors, triggering factors, and maintaining factors [287]. In sports disciplines requiring specific body mass and emphasizing physical image (e.g., gymnasts, combat sports athletes, middle- and long-distance runners, road cyclists, etc.), the pressure for weight loss during tapering for competition could be a precipitating factor for the manifestation of inadequate and sometimes extreme dietary behaviors [288,289]. This pursuit of the ideal body is deeply linked to the propensity for sports to cause EDs, particularly bulimia nervosa, as athletes are not guided towards a healthy diet but rather urged to achieve a standardized, “model body” [289]. Moreover, the psychological stress associated with sports competition and the focus on performance-related goals over psychological needs can increase the risk of developing an ED [290]. A relevant characteristic of this risk is the motivational focus that athletes bring to their sports participation [290]. Intrinsically motivated athletes engage in sports for the inherent enjoyment, satisfaction, and pleasure of the sport. These athletes are driven by feelings of enjoyment, satisfaction, and competence when learning new skills, mastering difficult techniques, or experiencing sport-related sensations [290]. Extrinsically motivated athletes are influenced by external factors of their participation, such as winning, obtaining material rewards, seeking public approval, or avoiding disapproval from others. Rather than valuing the sport itself, these athletes tend to participate more for the outcome than the process [290]. In the same study, it was explained that it is not the sport itself but the way an athlete relates to the specific challenges associated with the sport that may entail a risk or resilience of an ED. These findings speak to examining the quality of motivation when assessing the risk of EDs among athletes in high-risk sports contexts.

### 15.2. Coping and the Psychological Burden of High-Performance Competition

Participation in sports offers numerous benefits to athletes’ health, self-esteem, confidence, skill development, and social functioning [290]. However, negative experiences in the sports environment are not uncommon [291]. Since the beginning of the decade, there has been increased attention to health and mental illness in elite sports. Among elite athlete populations, the prevalence of mental illnesses varies annually between 5% and 35% [292]. In this context, Saura et al. [293] focused on identifying the main psychological problems associated with elite athletes, conducting research on high-level French competitors. They observed that 17% of these athletes suffered from generalized anxiety disorder and 4.2% had unspecified eating disorders. Additionally, 20.2% of female athletes exhibited at least one psychopathology, which is higher than the 15.1% ratio of male athletes (related to anxiety, depression, sleep problems, or self-harming behaviors). Athletes in “aesthetic” sports showed a higher percentage of generalized anxiety, at 38.9%, compared to those in high-risk sports, which stood at 3% [293].

Athletes competing at the elite level are exposed to unique risk factors for health and mental illness, such as intense performance demands, rigorous training schedules, media attention, injuries, and potential elimination [292]. These factors adversely affect the athlete’s commitment, performance, and well-being, contributing to negative outcomes, like overtraining and burnout, self-esteem deterioration, and affective disorders, such as anxiety and depression [291]. Additionally, there are factors that predominantly affect female athletes, such as non-acceptance in certain cultures, inequality in training opportunities, limited financial support, sexualization, stereotypes of sexuality, and societal and personal expectations surrounding traditional gender roles [292]. According to Burns et al. [293], achieving a world-class, podium-level performance is multidimensional, involving not just “talent” and training but also key psychosocial factors that athletes consider of utmost importance. These factors include psychological skills and attributes, interpersonal relationships, performance factors/strategies, and lifestyle practices [294]. From this perspective, success, both within the sport and in transition out of it, requires a holistic approach to athlete development that includes a complementary combination of well-being, lifestyle practices, performance strategies, psychological attributes, education, and supportive interpersonal relationships.

### 15.3. Female Athlete Triad

The female athlete triad is recognized as a neuroendocrine and nutritional syndrome (energy requirement) wherein a woman (regular physical exercise practitioner or a medium-to-high performance athlete) experiences deficiencies or issues in energy requirements (balance of utilization and replenishment of macronutrients and micronutrients in the diet), menstrual cycle dysfunction (including the ovarian cycle and functionality of the hypothalamic–pituitary axis), and altered bone mineral density [295,296]. In sports with specific total body mass (weight) and aesthetic prototype requirements, body weight control becomes highly significant for athletes [296]. Thus, these athletes not only confront societal beauty ideals but also face the bodily characteristics established by their sports discipline [287], such as in artistic gymnastics, combat sports, etc. In these disciplines, athletes are subjected to early concerns about their appearance (abdominal contour, waist-hip circumference, etc.), weight fluctuation, body composition (fat percentage), and, particularly, dietary habits for maintaining or losing weight [289]. The perception of body weight is also influenced by coaches, competition judges, media, and family members. Consequently, this situation may lead to body dissatisfaction and indicate inappropriate dietary behaviors in gymnasts [289]. However, for some athletes, the struggle with weight control can lead to serious and lasting consequences [296]. In female athletes, some of these consequences are multisystemic (affecting various body systems/organs). Additionally, the triad also manifests in male athletes with adverse metabolic, neuroendocrine, and reproductive consequences. The causes that predispose, precipitate, or maintain the “Triad” are multifactorial, including:

(a) Issues related to female physiology (hormonal regulation, neuroendocrine axis alterations, and menstrual cycle dysfunctions) and alterations in testosterone production in men;
(b) Eating behavior disorders (an inadequate diet);
(c) Intense or strenuous physical exercise, as well as the development of very demanding training cycles in terms of external and internal load programming.

This can lead to an energy deficit that may, in the long term, alter the female gonadotropic axis, causing menstrual irregularities and hypoestrogenism. Additionally, the lack of estrogens and insufficient nutrition (vitamin and mineral deficiencies) are responsible for decreased bone mineral density, accelerating the onset of osteoporosis [297,298].

Abnormal eating behaviors include restricting the intake of certain food groups, preoccupation with weight, bingeing and purging (self-induced vomiting and use of laxatives), excessive training, use of diuretics, and even the use of appetite-suppressing drugs. Consequently, the athlete presents with a low energy availability (LEA) that affects the functional energy requirements (at the basal level) of the body systems and the physiological and cognitive parameters involved in competitive performance in both men and women. While moderate and supervised exposures to low energy availability can be adaptive at certain points in the athlete’s multi-year programming, causing mild and quickly reversible changes in the biomarkers of various body systems signaling an adaptive distribution of energy and the plasticity of human physiology (e.g., improvement of body composition or a programmed period of intensive training or competition), this can be associated with acute health or performance benefits (e.g., increased relative VO_2_ max) [299]. In this context, adaptable LEA is usually a short-term experience with minimal (or no) impact on long-term health, well-being, or performance. However, prolonged exposure to a LEA can be problematic, causing a major and potentially persistent alteration of various body systems, often with signs and/or symptoms, and representing a maladaptive response. Furthermore, problematic LEA is associated with eating disorders in high-performance athletes.

### 15.4. The RED-S Model

The conceptual models of RED-S (relative energy deficiency in sport) highlight the detrimental effects on the functionality of body systems and athletic performance. Various factors can predispose and precipitate problematic LEA (low energy availability) in sports, such as (a) personal characteristics (gender, age, metabolic–endocrine alterations like estrogenic disruption, genetic alterations, etc.), (b) physical performance characteristics of the competition, (c) the athlete’s clinical history, (d) nutritional habits and beliefs about various food groups, and (e) psychological disturbances due to high exposure to environmental (sociocultural) stress, among others.

The latest consensus on LEA and RED-S, endorsed by the International Olympic Committee [299], presents the scientific and methodological foundations for the comprehensive approach to various practices by athletes related to low carbohydrate availability (LCA), LEA in the development of RED-S, and the onset of overtraining syndrome (OTS) that triggers a cascade of alterations in the biomarkers’ responses to the problem of LEA in the development of RED-S. Among the main consequences of RED-S evident in women are the deterioration of reproductive function, alteration of LH concentrations or its pulsatility, reduction in estrogen and progesterone, reduction of testosterone, primary amenorrhea, oligomenorrhea, secondary amenorrhea (FHA), luteal phase defects/deficiency, and anovulatory cycles, among others. In this context, women with different levels of physical condition and lifestyle can benefit from participation in physical-sport activities, supervised physical exercise, and sports development in terms of performance. However, pubescent, adolescent, and adult women, whether physically active or high-performance athletes, are not exempt from the risk of developing the female athlete triad (TF) [300].

Therefore, LEA and its multifactorial consequences should be addressed and evaluated as an ongoing process in which the practitioner and/or athlete understands, faces, and commits to comprehensive treatment (endocrine, psychonutritional, and sports) that allows for healthy and sustainable advancement in physical performance. Sports and physical exercise (EF) professionals need to understand what it involves and how to promote the prevention and/or recovery of the athlete through proper training programming. Of particular interest is the multidisciplinary approach in the clinical care of athletes with LEA and the significant role that nutritional care plays in preventing EDs and related complications [283]. Key nutritional therapies require knowledge about the nutritional requirements for the individual’s life stage, nutritional rehabilitation treatments, and modalities to re-establish normal eating patterns [283].

## 16. Practical Applications

According to the benefits and implications of physical activity and health in physiological and psychological areas [301,302,303,304,305,306,307,308,309], many practical applications are proposed. There are some more important factors to take into account:

○ Enhancing cognitive function through exercise: Given the demonstrated neurobiological impact of physical activity on the brain, particularly the hippocampus, training regimes can be scientifically tailored to enhance cognitive functions. Studies have shown that physical activity facilitates neurogenesis and the release of BDNF, which are critical for memory and learning. Therefore, exercise programs, especially for older adults or those at risk of cognitive decline, can be structured to incorporate aerobic exercises that are known to stimulate hippocampal activity.
○ Physical activity as a tool for emotional regulation: The modulation of mood and stress by physical activity, underscored by the release of neurotransmitters such as endorphins, serotonin, and dopamine, suggests that structured physical activities can be effectively used as non-pharmacological interventions in managing emotional disorders. Schools and workplaces can integrate physical exercise into their routines to harness these benefits, potentially reducing the prevalence of stress and anxiety-related issues.
○ Resilience building through sports participation: The development of mental fortitude and resilience through sports is a key area for practical application. For individuals battling psychological challenges, participation in sports can provide a structured environment for not only physical strengthening but also for enhancing psychological resilience. This is particularly beneficial in therapeutic settings, where sports activities can be used as part of treatment plans for mental health disorders, offering a holistic approach to recovery.
○ Integration of mindfulness in athletic training: The incorporation of mindfulness principles in sports presents a significant potential for enhancing athlete well-being and performance. Mindfulness practices, when melded with physical training, can aid in reducing performance anxiety, improving focus, and fostering a balanced emotional state. This can be particularly beneficial in high-stress competitive sports environments.
○ Sports practices in clinical settings for mental health disorders: The therapeutic potential of sports practices in clinical settings, especially for disorders like MDD, anxiety, and bipolar disorder, is substantial. Tailoring physical activities as part of the therapeutic process can offer a complementary approach to traditional mental health treatments, aiding in symptom management and overall well-being.
○ Fostering social dynamics through team sports: The psychological benefits of teamwork and camaraderie in sports can be leveraged to improve social skills and group dynamics. This is particularly relevant in educational settings, where team sports can be used as a platform for teaching cooperation, communication, and collective problem solving.
○ Optimizing environmental influences for psychological well-being: The impact of different settings on psychological outcomes in physical activities suggests that both natural and built environments should be optimally utilized. For instance, promoting outdoor activities can leverage the therapeutic effects of nature, while well-designed indoor environments can enhance motivation and mental well-being.
○ Addressing and preventing exercise addiction: Awareness of the potential for exercise addiction necessitates a balanced approach in training and fitness programs. Fitness professionals and sports psychologists should be equipped to identify the early signs of exercise addiction and provide guidance to ensure a healthy balance between physical activity and other life aspects.
○ Cultural sensitivity and gender inclusivity in sports programs: Designing sports programs with a deep understanding of cultural and gender diversity can lead to more effective and inclusive training and therapeutic interventions. This requires an acknowledgement of the varied cultural values and beliefs about physical activity and a sensitivity to gender-specific health needs and preferences in sports participation.
○ Mental strategies for performance enhancement: Incorporating mental strategies, such as visualization, goal setting, and cognitive–behavioral techniques, into sports training can enhance performance. This approach is justified by the understanding that psychological factors play a crucial role in athletic performance. Developing mental training programs that complement physical training can offer athletes a comprehensive toolkit for success.

## 17. Conclusions

This review has comprehensively explored the intricate relationship between sports, psychology, and mental health, underscoring the profound impact of physical activity on psychological and cognitive well-being. Through a multidimensional lens, it has been established that sports extend beyond physical exertion to become a potent tool for emotional regulation, resilience development, cognitive function enhancement, and the treatment of various psychological conditions. Neurobiologically, physical activity has been shown to induce significant neurochemical and neurophysiological changes, enhancing neurogenesis and synaptic function, particularly in brain regions like the hippocampus. This underscores the critical role of sports in enhancing memory, learning, and brain plasticity, even as a means to combat age-related cognitive decline.

In terms of mental health, physical activity emerges as an effective medium for emotional regulation and stress management. Regular exercise not only improves mood and reduces symptoms of disorders such as depression and anxiety but also strengthens social skills and community sense. Furthermore, the incorporation of mindfulness practices in sports offers a pathway to improved concentration, reduced performance anxiety, and overall well-being. The social aspect of sports, especially through teamwork and camaraderie, plays a vital role in developing interpersonal skills and promoting a sense of belonging. Environmental implications, such as the influence of different settings on psychological outcomes, are also pivotal, highlighting how natural and designed environments can optimize the psychological benefits of physical activity.

Considering cultural and gender perspectives is crucial for effectively understanding and applying sports and psychology principles across diverse populations. Acknowledging these perspectives enriches our understanding of how sports can be tailored to meet the specific needs of various groups, enhancing inclusivity and effectiveness in sports-related interventions. Thus, this discourse reinforces the importance of integrating physical and psychological approaches in sports, demonstrating their combined efficacy in promoting overall mental health and well-being.
